# Mental disorders, brain disorders and values

**DOI:** 10.3389/fpsyg.2014.00130

**Published:** 2014-02-17

**Authors:** Anneli Jefferson

**Affiliations:** ^1^Institute for Neuroscience and Medicine, INM8, Research Centre JülichJülich, Germany; ^2^Department of Philosophy, King's College LondonLondon, UK

**Keywords:** mental disorder, brain disorder, dysfunction, objectivity, normativity

The debates about the normativity of mental disorders and about the distinction between somatic and mental disorders have long been closely linked. This is very obvious in Szasz, who claims that there can only be brain disorders, no mental disorders and that so-called mental disorders are really problems in living. The implication of the latter claim is that people who have mental disorders are really people whose behavior and emotions depart from societal expectations. One might therefore be tempted to think that the normativity claim and the claim that mental disorders are really brain disorders stand and fall together. This is indeed what Stier claims. “Because of the normative nature of psychiatry, mental disorders cannot be completely reduced to neuronal or molecular processes.” (Stier, 2013, p.8)

But how close is the link between normativity and irreducibility really? I agree with Stier that ascriptions of mental disorders are intrinsically normative, and that what counts as a mental disorder has to be decided at the mental rather than at the brain level is also correct. However, the normativity claim and the claim that physicalism does not imply that all mental disorders are brain disorders can and should be separated for two reasons: First, we do not need the appeal to value judgments to justify the importance of the mental level in description and explanation. Second, we need to invest significant normative judgments in any kind of ascription of disease or disorder, not just in the range of the mental.

## Mental disorders and brain disorders

As Schramme (this issue) and others rightly point out, we cannot do without the mental level of description because the decision what counts as dysfunctional is made at the level of behavior and mental states. This holds even for disorders which are commonly understood as brain disorders, such as for example Alzheimer's. Alzheimer's counts as a disorder because of the problems with memory it is associated with. The importance of the mental level of description secures its continued relevance: “The claim that all instances of S have the property of realizing a disordered neurophysiological process is only possible at the psychological level of explanation” (Schramme, 2013, p. 5). It is therefore conceivable that we might end up being able to identify the physiological correlates of various mental disorders while still classifying these as mental disorders, as what marks these conditions and their corresponding brain states as disordered is a psychological defect.

One might think that the fact that mental disorders are physically based implies that mental disorders are *ipso facto* brain disorders and that what counts as a brain disorder becomes dependent on what counts as a mental disorder. In this way, the close link between mental disorders and brain disorders would be retained, but the concept of brain disorder would become dependent on that of mental disorder. But this inference is not licensed. It presupposes that there cannot be separate criteria for what counts as malfunction in the brain and what counts as a malfunction in the mind and that patterns of dysfunction can be pursued all the way down. However, this is by no means conceptually necessary. As has been pointed out in the literature, physicalism does not entail that where there is mental dysfunction, there is always a physical dysfunction. The computer analogy is sometimes invoked to illustrate the point that just as software problems do not imply hardware problems mental problems do not necessarily imply corresponding physical problems. As Boorse puts it “Whether and how a computer program, or a mental state, is dysfunctional need not be evident from any of its physical properties” (Boorse, 1976, p. 68).

This is not to say that there cannot be a close link between what is labeled as psychological dysfunction and what gets specified as brain dysfunction. While there are some clear cases of brain problems such as lesions which are specifiable without reference to the mental level, some of our conceptions of brain dysfunction are derived from the psychological level, rather than from independent conceptions of what counts as a brain malfunction. An example for this is the fact that the anomalies in the functioning of the amygdala found in psychopaths are labeled as dysfunctions because of the mental and emotional impairments they are associated with.

To summarize, whether dysfunction at the mental level is best described as dysfunction at the brain level is an empirical issue and it is by no means clear that very strong correlations can always be established. It may well be that in some cases, the way a certain disorder is realized in the brain is so disparate that no explanatory value is achieved by labeling the mental disorder in question as a brain disorder. Whether something should count as a brain disorder or a mental disorder will depend not on considerations regarding physicalism and the ultimate nature of the mind-brain relation but on what is explanatorily primary, the level of the mental or that of the physical. This is not a new thought, and it has been forcefully argued for in the context of the autonomy of the special sciences (cf. Fodor, 1974). But it is worthwhile reminding people that diagnoses of mental and brain disorders cannot simply be inferred from our ontological commitments but serve the purpose of explaining and treating these disorders. The importance of the first point has been stressed by Schramme and Stier, I hope to have helped shed some light on the latter.

## Normativity

I now want to turn to the normativity of mental disorders, a point Stier is particularly focused on. What he has in mind is not merely that all conceptions of disorder are at least minimally normative because they make reference to such notions as dysfunction and “correct functioning.” Rather, Stier endorses what he calls the stronger claim that psychiatry is guided by social, moral, cultural and other norms. That psychiatry is de facto a value laden discipline and that psychiatric diagnoses are to an extent dependent on the values that practitioners in the field and the surrounding society endorse is undeniable. I take it that Stier does not merely intend this as a true but fairly innocuous description of psychiatric practice. Rather, to have any philosophical bite, the claim must be that this is an essential feature of psychiatry which cannot be given up (see Rüther's comment in this issue).

One might think that subjectivity in norms is unproblematic if there are no objective values in the first place. According to this line of thought, a subjective or culturally relative diagnosis could only be wrong if there was an objective standard against which it could be measured as wrong. If this is not the case, then there is nothing troublesome in the difference of values which lead to different conceptualizations and classifications of disorder. However, I do not believe that a strongly relativist perspective is internally coherent. It would assume that the cultural relativist would have to concede that what is a disorder in their own society is not in another. For example, a homosexual would change from being disordered to being healthy simply by moving from a culture where homosexuality is seen as a mental disorder to one where it is not. But it does not seem credible to me that anyone would actually concede this degree of relativity for their own ascriptions of disorderedness. A certain amount of vagueness is of course unavoidable, but when we ascribe mental disorders, we try to get it right and worry that we may end up wrongly labeling something as a disorder.

The knowledge that ascriptions of mental disorder are in fact often culturally biased is troublesome because diagnosing someone with a mental disorder has far reaching practical consequences. If people can be sectioned because of acute mental disturbance, we do not want decisions as to what constitutes mental disturbance to be culturally arbitrary. Otherwise, social deviance could be labeled as mental disorder and used as a way of stigmatizing or discrediting people, or even withdrawing their personal freedom. For all of its weaknesses, the general definition of mental disorders in the DSM tries to address this issue, stating that “Socially deviant behavior (e.g., political, religious, or sexual) nor conflicts that are primarily between the individual and society are mental disorders unless the deviance or conflict results from a dysfunction in the individual, as described above”(American Psychiatric Association, 2013, p.20). Unfortunately, this does not really solve the problem, as it brings us back to the question what a dysfunction is. There needs to be a standard according to which we judge whether calling a certain condition pathological is valid or not.

The consequence some writers, in particular Christopher Boorse, have drawn from this is that we need to purge psychiatry of values and endorse a scientific notion of disease, whereby any residual normativity can be explained in biological terms. He proposes that we can define disease in terms of dysfunction and dysfunction in terms of deviation from normal species-typical functioning. Normal functioning is described as typical contribution to survival and reproduction. The problem with this type of account is that it only seemingly avoids normativity. Unless it is a purely statistical notion, talk of dysfunction presupposes that there is something the mind or body should be doing, not merely something it normally does. A statistical notion of dysfunction and pathology is too thin to be useful for medical practice. This does not mean that it cannot be extremely useful in describing various conditions. But arguably, the move from describing something as anomalous to describing it as a dysfunction always requires a commitment as to how something is supposed to function which goes beyond observations on what is statistically normal.

Fortunately, we need not assume that being evaluative and being objective are mutually exclusive unless we are given a convincing argument to the contrary. Rather, we can look for some specification of harms ensuing from anomalous mental conditions which we can use to justify that a certain condition is indeed a mental disorder. An example for such an attempt is Gert and Culver's proposal for a definition of mental disorder drawing on the list of harms given in the DSM IV TR. They point out that the list only contains states considered to be harms cross-culturally, such that there is an objective, broadly shared notion of harm. “The agreement of rational persons in all societies about the universality of the basic harms is extremely important, for it establishes the objectivity of the concept of a disorder” (Gert and Culver, 2004, 421f.). It should be pointed out that, just as de facto disagreement and subjectivity does not automatically entail the subjectivity of all value, neither does de facto agreement automatically establish objectivity. Nevertheless, a theory of value does well to take the things people actually value and agree on as a starting point.

In conclusion, ascriptions of disorder are normative, but we should strive for objectivity in our evaluations. Furthermore, there is no need to fear that advances in the brain sciences will make the level of the mental redundant.
